# Patients with Retinitis Pigmentosa May Have a Higher Risk of Developing Open-Angle Glaucoma

**DOI:** 10.1155/2022/9719095

**Published:** 2022-06-22

**Authors:** Man-Chen Hung, Yu-Yen Chen

**Affiliations:** ^1^Department of Medical Education, Taichung Veterans General Hospital, Taichung 407, Taiwan; ^2^School of Medicine, National Yang-Ming University, Taipei 112, Taiwan; ^3^Department of Ophthalmology, Taichung Veterans General Hospital, Taichung 407, Taiwan; ^4^Wilmer Eye Institute, Johns Hopkins University, Baltimore 21287, Maryland, USA; ^5^Institute of Public Health and Community Medicine Research Center, National Yang-Ming University, Taipei 112, Taiwan; ^6^School of Medicine, Chung Shan Medical University, Taichung 402, Taiwan; ^7^National Chung Hsing University, Taichung 402, Taiwan

## Abstract

**Background:**

Retinitis pigmentosa (RP) is the most common retinal hereditary dystrophy, which can lead to blindness if it progresses. Similarly, open-angle glaucoma (OAG) is a genetic disorder. The similarities in genetic variants and pathophysiology between RP and OAG have been reported. We sought to explore whether patients with RP have a significantly higher risk of OAG development.

**Methods:**

We enrolled patients with RP into the RP group through Taiwan's National Health Insurance Research Database from 2001 to 2013; we included a comparison group of 1 : 4 age- and gender-matched individuals without RP. We performed a Cox regression analysis to estimate the crude and adjusted hazard ratios (HRs) for OAG. We adjusted the following confounders in the Cox regression model: age, gender, diabetes mellitus, hypertension, and chronic kidney disease.

**Results:**

We enrolled 6,223 subjects with RP and 24,892 subjects for comparison. The mean age of the cohort was 49.0 ± 18.1 years. The RP group had significantly higher percentages of diabetes mellitus, hypertension, and hyperlipidaemia. The cumulative incidence of OAG in patients with RP was 1.57%; this was significantly higher than that in the comparison group (0.58%, *p* < 0.0001). On univariate Cox regression analysis, the hazard of OAG development was significantly greater in the RP group than in the comparison group with an unadjusted HR of 2.86 (95% confidence interval, 2.21–3.70). The increased risk persisted after adjusting for confounders (adjusted HR = 2.86; 95% CI, 2.21–3.70).

**Conclusions:**

This nationwide population-based cohort study showed that people with RP are at a significantly greater risk of developing OAG than individuals without it.

## 1. Introduction

Retinitis pigmentosa (RP) is a genetic disorder involving the breakdown and loss of photoreceptor cells [1]. Patients may first present with night vision loss, followed by peripheral vision field defects, optic nerve dysfunction, and blindness [2]. The characteristics of the fundus in RP include retinal arteriole attenuation, intraretinal pigmentation, and waxy pallor of the optic disc [3]. Open-angle glaucoma (OAG) is also characterised by the atrophy of the optic disc and loss of visual field. In addition to the elevated intraocular pressure, specific genetic variants may contribute to the pathogenesis of OAG [4–6].

Greenstein et al. reported that RP and OAG had similar pathways in cone vulnerability, including S and M cone losses [7]. Besides, RP presents with a reduction of blood flow [8], which is similar to the vascular dysfunction in OAG [9, 10]. Therefore, the two diseases might be associated due to their common pathophysiology.

However, very few studies have investigated the relationship between RP and OAG [11, 12]. A clinical analysis in China found that 32 in 1,400 patients with RP (2.3%) had glaucoma (30° angle-closure glaucoma; 2 OAG) [12]. We could not fully understand the relationship between RP and OAG due to the scarcity of previous literature and the rare occurrence of glaucoma among RP. In our study, we hypothesized that patients with RP had a higher risk of developing OAG. We used the National Health Insurance Research Database (NHIRD) in Taiwan to enroll a larger number of patients with RP and follow the incident event and risk of OAG.

## 2. Materials and Methods

### 2.1. Data Source

The National Health Insurance (NHI) program of Taiwan covers the healthcare services of 99.82% of residents (a total population of 23 million). The NHIRD maintained by the National Health Research Institutes of Taiwan is released for research purposes and contains demographic data for all NHI enrollees and health care, medical prescription, and surgical management data for ambulatory and in-hospital patients in Taiwan. Diagnoses are registered according to the International Classification of Diseases, Ninth Revision, Clinical Modification (ICD-9-CM) codes. We used NHIRD data on health care claims for the entire population from 1996 to 2013 for this retrospective cohort study. To ensure confidentiality, the National Health Research Institutes (NHRI) encrypted the personal information of all patients in the database prior to releasing data for academic use. Written informed consent was waived according to the rules of the Institutional Review Board. This study was approved by the Ethical Committee of National Yang-Ming University Hospital. (no. 2015A018).

### 2.2. Inclusion and Exclusion Criteria

We selected patients diagnosed of RP (ICD-9-CM code 362.74) from the NHIRD from January 1, 1996, to December 31, 2013. RP was confirmed by ophthalmologists through a well-acknowledged standard diagnostic protocol with retinal bone-spiculae pigments, arteriolar attenuation, and waxy pallor of the optic disk under indirect ophthalmoscopy [5]. Visual field examination revealed a mid-peripheral visual field defect or central island. Electroretinogram showed a reduced rod and cone response and delay of time [5, 13]. Cases of RP variants may be missed (e.g., RP sine pigmento and sector retinitis pigmentosa); therefore, optical coherence tomography is applied to confirm the loss of the photoreceptors and the retinal pigment epithelium. To ensure that the enrolled patients with RP were newly diagnosed during our study period, those with RP diagnosed before the end of 2010 were excluded (January 1, 2011, to December 31, 2013). The index date was defined as the date of the first RP claim.

Thereafter, we randomly selected individuals who had never been diagnosed of RP as a comparison group at a ratio of 1 : 4 and matched them with the RP group in terms of age, gender, and index year (year of enrolment). All eligible RP subjects and comparisons had never been diagnosed with OAG before the index date.

## 3. Definition of Outcome

We compared whether the two groups developed subsequent OAG (ICD-9-CM code 365.1) confirmed by ophthalmologists by following up to the end of 2013. Patients diagnosed with OAG should have a glaucomatous visual field defect or glaucomatous optic neuropathy with an open angle [14]. Although elevated IOP is a risk factor of glaucoma, it is not among the definition of OAG in our study. OAG in our study included primary hypertensive (ICD-9-CM code 365.11) and normotensive types (ICD-9-CM code 365.12). To derive a pure impact of RP on glaucoma and diminish factors that could confound our analysis, we excluded patients with ocular surgery or trauma because glaucoma may develop after these events. Besides, we focused our outcome on primary OAG, excluding secondary glaucomas such as exfoliative glaucoma, pigmentary glaucoma, and steroid-induced glaucoma. Since double arcuate scotoma of OAG can mimic the ring midperipheral scotoma of RP, the progression of the visual field defect in RP patients alerts us to the possibility of glaucoma.

### 3.1. Identification of Comorbidities

We identified comorbidities, which confound the association between RP and OAG [13] as covariates to be adjusted in subsequent statistics. The included comorbidities were diabetes mellitus (ICD-9-CM code 250), hypertension (ICD-9-CM code 401–405), hyperlipidaemia (ICD-9-CM code 272), and chronic kidney disease (ICD-9-CM code 585).

### 3.2. Statistical Analysis

After investigating the demographic/clinical characteristics of RP and the comparison cohorts, we computed the group difference using the two-sample *t*-test for continuous variables and the chi-squared for categorical variables.

We used the Cox proportional hazard model to calculate the hazard ratio (HR) and 95% confidence interval (CI) for the occurrence of OAG according to each variable in the univariate and multivariate analyses. Covariates adjusted in the regression analysis were age, gender, diabetes mellitus (DM), hypertension (HTN), hyperlipidaemia, and chronic kidney disease. To avoid immortal time bias, we considered comorbidities as time-dependent variables.

All analyses were performed using SAS version 9.3 (SAS Institute, Cary, NC, USA). A *p* value < 0.05 was considered statistically significant.

## 4. Results

### 4.1. Demographic and Clinical Characteristics of the Study Sample

In this study, we enrolled 6,223 subjects in the RP group and 24,892 matched comparisons.  Table 1 displays the demographics of the two groups. The mean age of the overall cohort was 49.0 years with a standard deviation of 18.1 years. Females accounted for a slightly higher proportion than males (51.3% vs. 48.6%). The two groups were well matched for age and gender.

Compared to the comparison group, the RP cohort had significantly higher percentages of patients with DM (20.4% vs. 17.7%, *p* < 0.0001), HTN (38.5% vs. 36.3%, *p*=0.001), and hyperlipidaemia (28.6% vs. 25.7%, *p* < 0.0001). However, the prevalence of chronic kidney disease between the two groups was similar (5.2% vs. 5.6%, *p*=0.26). During the study period, the cumulative incidence of OAG was 1.57%, which was significantly higher than that of the controls (0.58%) (*p* < 0.0001).

### 4.2. Univariate and Multivariate Analyses by the Cox Regression Model

Table 2 shows the HRs of OAG during the 13-year study period calculated with univariate and multivariate Cox regression models. On univariate analysis, RP, age, DM, HTN, and hyperlipidaemia carried a significantly higher risk of developing OAG. The unadjusted HR for OAG was 2.86 times greater in the RP group than that in the comparison group (95% CI: 2.21–3.70; *p* < 0.0001).

RP was still significantly associated with an increased risk of developing OAG (adjusted HR = 2.83, 95% CI: 2.19–3.66) after adjustment for covariates. In addition, age was a significant risk factor for RP on univariate and multivariate analyses. The adjusted HR for patients over 60 years was 2.45 relative to those younger than 40 years (adjusted HR = 2.45, 95% CI:1.63–3.68). However, after adjusting the covariates, neither gender nor comorbidities remained a significant risk factor for OAG.

## 5. Discussion

This 13-year population-based study from the Taiwan NHIRD showed a significantly higher cumulative incidence of OAG in the RP group compared to age- and gender-matched controls. After adjustment for age, gender, and comorbidities in a multivariate Cox regression analysis, RP still had a significantly greater risk of incident OAG.

### 5.1. Prevalence and Age of Onset for RP

The worldwide prevalence of RP varies from 0.014% to 0.04% [15]. RP is rare and the prevalence depends on ethnicity as shown in the latest Asian study; the prevalence of RP was 0.03% in Malay, 0.06% in Indians, and 0.09% in Chinese [16]. Our study revealed that the prevalence of RP was 0.027% (6223 in 23 million people) in Taiwan, which is similar to that in another Taiwanese cohort in Ko's study (0.0382%) [17].

Concerning the age of onset for RP, several studies have shown its variation with ethnicity and genes [1]. According to Ko's study, the average age when patients are first diagnosed with RP is 51.1 years, which is similar to the result in our study result (49.0 years) [17]. However, another study in Japan found that RP was diagnosed at a mean age of 35.1 years [18].

### 5.2. RP and OAG

According to a large cohort study in China, the prevalence of glaucoma in patients with RP was 1.6% [19]. In our 13-year population-based study, we found that RP was significantly associated with incident OAG, which may be due to their collaborative pathogenesis.

First, the S and M cone losses in patients with RP and OAG were similar, which could be due to hypoxia [20]. Hypoxia might induce neovascularization in RP leading to abnormal autoregulation of retinal blood flow [3], thus increasing the susceptibility to glaucomatous damage [4,9].

Second, both RP and OAG have similar gene mutations [20]. Heterozygous mutations in human RP-PRPF genes are found to be associated with defects in pre-mRNA splicing, leading to RP [21, 22]. Likewise, Micheal et al. identified that pathogenic variants in the PRPF8 gene are associated with OAG [23]. Mutation of the retinitis pigmentosa GTPase regulator (RPGR) gene is another mutation present in RP and OAG. Once RPGR gene mutation occurs, the dysfunction of interaction between RPGR and RPGR-interacting protein 1 (RPGRIP1) finally leads to RP type 3 (RP3) [24]. Fernández–Martínez et al. figured that variants of RPGRIP1 and impairment of the interaction of RPGRIP1 with different proteins may also result in glaucoma [25].

### 5.3. Strengths and Limitations

Our study is the first to investigate the association between RP and OAG on a population-based scale. One of our strengths is that we had accurate information owing to the comprehensiveness and completeness of NHIRD, in which demographic data, diagnoses, examinations, and therapies are recorded and confirmed. The National Health Administration routinely checks medical charts to ensure that patients have correct diagnoses. Besides, we enrolled patients from the NHIRD from January 1, 2001, to December 31, 2013, which was the longest study period known. Furthermore, our study not only investigated the association between RP and OAG but also adjusted the impacts of the confounders. Therefore, the significant association between RP and OAG in our study was likely a real phenomenon.

One limitation of our study is that the clinical diagnosis of some cases of RP, especially RP variants, may be missed. Fortunately, we have used a series of thorough examinations such as fundus ophthalmoscopy, visual field, electroretinogram, and optical coherence tomography to increase the accuracy of the diagnosis. Another limitation of our study is the lack of genetic sequencing or laboratory data. Further studies will be needed to combine the Taiwan Biobank with the NHIRD to elucidate whether specific genes or biochemical profiles may explain the relationship between RP and OAG.

## 6. Conclusions

Our study concluded that people with RP have an increased risk of developing OAG. Therefore, ophthalmologists should be aware of the possible risk of developing OAG in patients with RP and pay more attention to the changes in the optic nerve during the regular follow-up.

## Figures and Tables

**Table 1:** Characteristics of the study subjects.

Variable	RP group	Non-RP group	*P* value
	*n* = 6223	*n* = 24892	
	*n* (%)	*n* (%)	
Age (years) (mean ± SD)	49.0 ± 18.1	49.0 ± 18.1	0.97

Age (categorical)			
<40	1893 (30.4)	7572 (30.4)	1.00
40–60	2494 (40.1	9976 (40.1)	
≥60	1836 (29.5)	7344 (29.5)	

Gender			
Male	3047 (49.0)	12101 (48.6)	0.63
Female	3176 (51.0)	12791 (51.4)	

Diabetes mellitus			
Yes	1269 (20.4)	4396 (17.7)	<0.0001
No	4954 (79.6)	20496 (82.3)	

Hypertension			
Yes	2398 (38.5)	9036 (36.3)	0.001
No	3825 (61.5)	15856 (63.7)	

Hyperlipidemia			
Yes	1781 (28.6)	6404 (25.7)	<0.0001
No	4442 (71.4)	18488 (74.3)	

Chronic kidney disease			
Yes	323 (5.2)	1385 (5.6)	0.26
No	5900 (94.8)	23507 (94.4)	

FU period (years)	6.1 ± 3.7	6.4 ± 3.7	<0.0001

OAG during the FU period	98 (1.57)	144 (0.58)	<0.0001

RP: retinitis pigmentosa; SD: standard deviation; OAG: open-angle glaucoma. FU: follow-up. Data are presented as mean ± standard deviation or *n* (%).

**Table 2:** Analyses of risk factors for OAG in patients with and without RP.

Predictive variables	Univariate analysis		Multivariate analysis	
	Unadjusted HR (95% CI)	*p* value	Adjusted HR (95% CI)	*p* value
RP (yes vs. no)	2.86 (2.21–3.70)	<0.0001	2.83 (2.19–3.66)	<0.0001

Age				
<40	Reference		Reference	
40–60	1.79 (1.27–2.53)	<0.001	1.74 (1.21–2.49)	<0.01
≥60	2.56 (1.80–3.64)	<0.0001	2.45 (1.63–3.68)	<0.0001

Gender (male vs. female)	1.18 (0.92–1.52)	0.111	1.23 (0.95–1.58)	0.111
Hypertension	1.55 (1.20–1.99)	<0.001	1.02 (0.74–1.40)	0.914
Diabetes	1.48 (1.10–1.97)	<0.01	1.10 (0.79–1.53)	0.586
Chronic kidney disease	1.07 (0.62–1.83)	0.808	1.01 (0.66–1.60)	0.904

OAG: open-angle glaucoma; RP: retinitis pigmentosa; HR: hazard ratio; CI: confidence interval. In the multivariable analysis, all the other variables in the table are included for adjustment.

## Data Availability

The National Health Insurance Research Database (NHIRD) data used to support the findings of this study were supplied by Taiwan National Health Insurance (NHI) Bureau under license and cannot be made available in the manuscript, the supplemental files, or in a public repository due to the Personal Information Protection Act executed by the Taiwan government, starting from 2012. Requests for access to these data should be sent as a formal proposal to the NHIRD (http://nhird.nhri.org.tw) or by e-mail to wt.gro.irhn@drihn.

## Abbreviations

AHR: Adjusted hazard ratio
CI: Confidence interval
DM: Diabetes mellitus
FU: Follow-up
HTN: Hypertension
ICD-9-CM: International Classification of Diseases, Ninth Revision, Clinical Modification
NHI: National Health Insurance
NHIRD: National Health Insurance Research Database
NHRI: National Health Research Institutes
OAG: Open-angle glaucoma
RP: Retinitis pigmentosa
SD: Standard deviation
RP3: Retinitis pigmentosa type 3
RPGR: Retinitis pigmentosa GTPase regulator
RPGRIP1: RPGR-interacting protein 1.
